# What we get wrong about stress in sports psychology: an opinion

**DOI:** 10.3389/fpsyg.2026.1752653

**Published:** 2026-03-16

**Authors:** Dilshith A. Kabeer, Vinu W., Safad A. K., Karuppasamy Govindasamy, Balaji E., Logeswaran A. S., Rajkumar M., Shareef K. K., Aravind M. K., Pramitha M.

**Affiliations:** 1 Department of Physical Education and Sports, Pondicherry University, Pondicherry, India; 2 Department of Sports, Recreation and Wellness, Symbiosis International (Deemed University), Hyderabad, India; 3 Department of Physical Education, C.B.M. College, Coimbatore, India; 4 Department of Physical Education, Bharathiar University, Coimbatore, India

**Keywords:** athlete burnout, biopsychosocial model, psychophysiology, sport psychology, stress regulation

## 1 Introduction

For centuries, athletes have faced multiple forms of stress arising from training demands, competitive pressure, organizational environments, and broader life contexts. Historical models in sport first conceptualized stress through biomedical perspectives, viewing it primarily as a physiological response to environmental demands, building on Selye's General Adaptation Syndrome and later distinctions between sustress (inadequate stress), eustress (good stress), and distress (bad stress) as different states of challenged homeostasis (Lu et al., 2021). Subsequent psychological and transactional approaches shifted attention toward cognitive appraisal, emotion, and coping, examining how athletes interpret anxiety, experience emotions, and deploy coping strategies, as well as how these dynamic processes predict performance in competition (Meijen et al., 2020; Nogueira et al., 2025). More recent integrative frameworks, such as biopsychosocial and psycho-neuro-endocrino-immunological (PNEI) models, conceptualize stress as a dynamic interaction among biological, psychological, and socio-cultural systems, emphasizing allostatic load, distress–eustress balance, and lifestyle- and context-dependent variability in stress responses (Den Hartigh et al., 2024; Nuetzel, 2025). Importantly, PNEI frameworks explicitly acknowledge the pioneering work of Hans Selye as a foundational antecedent rather than an alternative theoretical model. Traditional PNEI literature emphasizes that Selye's conceptualization of stress as a systemic adaptive response represented a critical turning point beyond an exclusively reductionist biomedical perspective. This theoretical change enabled the development of the modern integrative models, which take into account the neural, endocrine, immune, and psychosocial aspects (Bottaccioli and Bottaccioli, 2005; Jackson, 2014). In line with these integrative points of view, a growing body of literature indicates that stress can be biologically entrenched through an epigenetic process in which environmental exposures can regulate the expression of genes without changing the DNA sequence. Empirical data based on human research suggests that stress during prenatal growth and early life is linked to both epigenetic changes relevant to stress control and mental health, thus representing intervals of increased biological plasticity and susceptibility (Xu et al., 2020; Zhou and Ryan, 2023). Notably, stress sensitivity through epigenetics is not confined to early life, where chronic psychosocial, occupational or traumatic stress during adulthood has also been associated with epigenetic alterations which could influence long-term stress responsiveness and psychological performance (Ochi and Dwivedi, 2023).

In spite of this theoretical development, a significant percentage of sport interventions still regards stress as a predominantly a mental issue and focuses on discrete changes in appraisal, emotion or coping without attention to physiological regulation, lifestyle, and organizational or cultural factors. This type of enduring conceptual incongruity promotes superficial and reactive activities that cannot adequately respond to cumulative allostatic load, chronic life and organizational stress, as well as its connections with burnout and performance instability among athletes (Cai et al., 2025; Ma et al., 2025). Modern stress-management interventions in sport are largely cognitive and emotional, and aim to intervene once issues arise, and not to prevent and regulate training periods. Therefore, the concept of stress remains poorly understood as an event that is mostly of a mental nature, with the lack of consideration of physiological control, social factors, and lifestyle-related factors (Souza-Talarico et al., 2016). This type of narrow framing creates incomplete systems of support, and sustained stress, burnout, and instability of performance. At the same time, the one-size-fits-all models of intervention are still in use, even though there is strong evidence to suggest that the outcomes of stress responses differ significantly across individuals and are influenced by biological, psychological, and contextual factors.

The current studies show that there is an enormous inter- and intra-individual inconsistency in stress reactivity and coping, contingent upon age, sex, genotype, epigenetics, health condition, as well as lifestyle (Pereira-Figueiredo et al., 2024). Hypothalamic–pituitary–adrenal (HPA) axis responses to psychosocial stress, particularly cortisol reactivity, show pronounced variability, while exercise and training studies consistently identify “high-responder” and “low-responder” patterns to standardized workloads, driven by baseline physiology and genetic predispositions (Mann et al., 2014; Zänkert et al., 2019). Previous exposure to stress also alters future psychophysiological response to exercise. Personality factors and coping mechanisms (e.g., trait anxiety, problem- vs. emotion-focused coping) are linked to unique psychobiological stress profiles, and social support and lifestyle resources could mitigate or enhance the impact of stress on health and performance (Soliemanifar et al., 2018; Sandvik et al., 2020; Jacoby et al., 2021). Furthermore, long-term stress phenotypes are influenced by early adversity and non-shared life experience and they are affected by gene-environment interactions. Nevertheless, many sport-stress interventions continue to ignore individual diversity, based on biology, sport demands, gender, disability, and cultural background, and follow standard approaches that cannot meet the unique needs of athletes (Guest et al., 2019). The conceptualization of stress as purely mental, consciously controlled and reactive as opposed to preventive can contribute to maladaptive stress processes, thus increasing the risk of burnout, injury and performance instability.

To this end, this opinion piece will attempt to refute the myths in sports stress management by outlining the major shortcomings of the current practice, such as the overdependence of cognitive approaches, a reactive and not a preventive approach, and the persistence of the one-size-fits-all approach. The article is placed within a biopsychosocial and psychophysiological theoretical framework arguing that stress is to be understood as a multisystem regulation process and not necessarily as a psychological disturbance. Instead of neglecting the current strategies, we promote more holistic, integrative, personalized and preventive approach which consolidates mental, physical and social resources. In the case of athletes, this orientation facilitates the emergence of effective stress-management skills which are, at the same time, interdisciplinary, preventive, and individualized.

## 2 Discussion

Despite decades of scientific research, we still have very little knowledge of stress in sports psychology. Transactional and cognitive–appraisal models reframed stress as a dynamic person–environment process and have long guided sport research on coping, emotion, and performance (Simpson et al., 2024). Newer biopsychosocial and PNEI models build upon this view by placing appraisal in allostasis and lifestyle regulation, with the recommendation of individualized, multisystem interventions (Tossici et al., 2024). Misconceptions have shaped narrow perspectives which led to designing insufficient interventions that often fail to address the full picture. These narrow perspectives have limited our perception of stress, creating disjointed responses to it, and preventing the understanding that stress is not only a burden but can also be a motivating factor to adaptation and development.

### 2.1 Why mind-based strategies falls short

The traditional perspective gives a picture that stress is only a psychological or emotional hurdle that has to be overcome. This view is based on precedent psychological paradigms, which advocate a mind over body approach, in which athletes received to use techniques like positive self-talk, visualization and reframing to cope with stress. These tools are valuable, yet they often ignore the deep physiological and adaptive aspects of our stress response. In modern conceptualizations, stress is understood as an actual or perceived threat to homeostasis that is triggered by the brain, and is mediated by coordinated responses of the autonomic nervous system, Hypothalamic-Pituitary-Adrenal (HPA) axis, and immunoendocrine circuits, with sustained stress resulting in allostatic overload among the various physiological systems (McEwen, 2017; Haykin and Rolls, 2021; O'Connor et al., 2021).

Competition pressure, chronic injury and heavy training loads have an impact on the body that cannot always be dealt with by mental techniques. This underlying discrepancy between the physiological realities and the psychological interventions reveals real consequences. Mind-based strategies may alleviate perceived stress but they do not always treat the underlying causes. Persistent muscle tension, disrupted sleep, increased cortisol levels, and weakened immunity might persist in athletes even after employing such strategies (Nuetzel, 2023; Renaghan et al., 2023). As an example, an athlete using reframing techniques to cope with pre-competition anxiety might report a lower subjective anxiety level, but the method might not reduce resting heart rate or enhance heart-rate variability (Sancinelli, 2023). This disconnection explains why mind-based approaches when implemented alone may lead to a decrease in perceived stress without addressing the underlying physiological burden. As soon as athletes realize that their mental work is not reflected in any visible physiological rewards, the disconnect may lead to burnout in the long-term, increased risk of injuries, and a feeling of complete powerlessness.

Chronic stress gets biologically implemented with long-lasting epigenetic and system-wide changes, but these changes are not irreversible. Cognitive methods might not be effective when applied alone in acute or chronic cases, but mind-based and psychotherapeutic approaches can alter stress biology and even epigenetic phenotypes. The evidence is in favor of the integrated brain-body approaches and not the strict dichotomy between mind and biology. The solution, therefore, is not to leave behind mental strategies but to enhance them by incorporating them. Stress control should be a collective effort which touches the brain and the body. Heart-rate-variability monitoring, biofeedback, breathing control and yoga are some of the techniques that offer a physical channel that helps to support mental mechanisms. These approaches move beyond the treatment of the symptoms to the whole stress-response system. Such integrative strategies, when done correctly, would be able to turn stress into a resource that would enhance resilience and sustain performance.

### 2.2 Coping isn't always a deliberate act

The modern sport psychology is starting to recognize coping as a multidimensional and dynamic regulatory system instead of a repertoire of consciously implemented, on-demand methods. Empirical studies with elite athletes show that coping is a flexible set of cognitive, emotional, and behavioral processes that changes depending on the nature, severity, and perception of a stressor such that athletes integrate and switch between different coping modes, instead of using independent strategies (Nicholls et al., 2016; Nuetzel, 2023). Coping automaticity is developed by using pressure-based training and repeated exposure to stress so that responses can be quick and context-specific when competition occurs (Low et al., 2023). Furthermore, coping has become interpersonal and systemic, and dyadic regulation in coach-athlete relationship and social-support network as a whole is also critical in stress regulation and mental health, thus highlighting the need to have integrated and coordinated support systems rather than individual cognitive interventions.

In the light of these risks, it is an urgent need to move beyond just imparting explicit coping skills to students, and to support implicit resilience via progressive and progressive exposure to stress and practice. These strategies are consistent with the current learning-and-adaptation models focused on embodied and procedural stress regulation. Moreover, the improvement of interaction and communication among support personnel is one of the pillars of establishing effective support systems that enrich personal and team-based coping resources, which have been proven to cushion stress and promote mental-health outcomes among athletes (Leprince et al., 2018). Finally, the combination of implicit and explicit coping resources and a socially supportive system facilitates the ability of athletes to deal with stress and stay well under the pressure of the competitions.

### 2.3 Prevention beats intervention

Stress is too often managed as a reactive intervention that only occurs when there are overt symptoms of anxiety, burnout, or decline in performance. This “firefighter” model ignores the need for proactive building of resilience as well as the development of stress regulation as a daily routine. Across sport, healthcare, and occupational contexts, evidence consistently shows that reactive, symptom-driven stress interventions are often too late and less effective than preventive, routine approaches that build resilience and sustain performance over time (Heath et al., 2020). In sport, embedding multimodal stress management and recovery monitoring (e.g., psychological skills, mindfulness, HRV, sleep, and psychometric indicators) within daily training structures enables early detection of under-recovery and targeted adjustment before maladaptation, injury, or performance decline occurs (Lopes Dos Santos et al., 2020; Adebisi et al., 2025). The best results are achieved when these techniques are combined into a comprehensive, well-rounded, multimodal system instead of designed as scattered add-ons (Lochbaum et al., 2022). These programs have been found to be most effective when they are continuous and incorporated into routine practice and not used as an on-off solution to issues arising.

Preventive models incorporate stress regulation in the normal training activities by monitoring recovery, workload, and psychological preparedness. These methods are especially relevant in light of new findings that have connected chronic stress exposure to physiological adaptation and maladaptation processes in the long term, including epigenetic processes that moderate the relationship between lifestyle and psychosocial stressors and biological regulation (Heidari et al., 2019; Nicolas et al., 2019). Chronic stress leads to allostatic load or overload and triggers long-term neuroendocrine, immune, and metabolic alterations which in turn may be maladaptive in case of non-prevention (McEwen, 2017). According to reviews, epigenetic mechanisms of repeated stress include changes in brain circuits, stress hormones, and immune functions that are linked to lifestyle and psychosocial stress throughout the lifespan (Bottaccioli et al., 2019). Cumulative stress is associated with accelerated epigenetic aging, partially buffered by resilience factors such as emotion regulation and self-control (Harvanek et al., 2021). Qualitative, life-course approaches have further illustrated how cumulative stress experiences shape athletes' stress regulation across development, reinforcing the value of preventive, long-term perspectives. Thiel's life-course work shows that athletes' stress responses are built cumulatively over time, so only early, continuous monitoring and proactive regulation can interrupt the maladaptive patterns that later interventions must merely manage rather than undo (Schubring et al., 2019). Proactive intervention using reactive instruments puts athletes at risk of avoidable performance and health deterioration due to ignoring the accumulated impacts of daily stressors and failure to address the underlying lifestyle determinants of sleep, nutrition, and recovery. The answer is a change toward a more preventative and holistic approach, whereby athletes would be more resilient, competent and psychologically fit. The interdisciplinary work of trainers, psychologists, and coaches will be necessary, where the stress management will be described not as a supplementary, but as an indispensable part of the athletic development.

## 3 The path forward: from reaction to regulation

The shortcomings of conventional, fragmented solutions imply that we should redefine our perception of the management of stress in sports. These methods fail to represent the dynamic, multi-component nature of stress responses, which occur within psychological, biological, and social systems. To address this limitation, a preventive, integrative, and personalized (IPP) approach aligned with biopsychosocial and psycho-neuro-endocrino-immunological (PNEI) models is warranted, as these frameworks conceptualize stress as a regulatory syndrome influencing allostatic load, lifestyle behaviors, and performance outcomes (Tossici et al., 2024). Within this framework, stress management is always proactive rather than reactive which integrates cognitive, physiological, and social aspects within a single protocol, and is personalized to individual stress constellations that differ among athletes, sports, and contexts. This does not mean that practical tools should be thrown away but rather re-packages their use in a co-ordinated system that would facilitate sustainable performance and long-term wellbeing of the athlete.

### 3.1 Stress as a unified system

Stress regulation can best be viewed as a combined process, which incorporates cognitive, embodied and feedback-based interventions instead of considering mind and body as distinct targets. Sport psychology evidence indicates that the combination of cognitive skills (e.g., imagery, self-talk, goal setting) with arousal-regulation and embodied practices (e.g., breathing, relaxation, mindfulness, yoga) has been shown to yield more improvements in attention, emotional regulation, physiological balance, and performance compared to single-component approaches (Bordo et al., 2025; Xie et al., 2025). This unification is further enhanced by biofeedback and neurofeedback, which directly connects mental strategies with immediate physiological reactions. Cognitive intent is operationalized as a coordinated mind-body system by converting it into measurable body change by adding indicators such as heart-rate variability, EEG activity, or galvanic skin response (Tosti et al., 2024; Bovolon et al., 2025). The combination of psychological training and physiological regulation will create a better state of the athlete to maintain performance and adjust well to the stressor.

### 3.2 Proactive over reactive

Stress management can be conceptualized as a multilevel, proactive regulatory process that extends beyond situational coping responses. While coping refers to the cognitive, emotional, and behavioral strategies mobilized after a stressor is appraised as challenging or threatening, stress management operates across stress triggers, stress intensifiers (e.g., appraisals and beliefs), and stress reactions over time, as outlined in multilevel models of stress regulation (Kaluza and Chevalier, 2016). From this system-level perspective, stress management also encompasses longer-term biological embedding mechanisms, including epigenetic processes, through which cumulative psychosocial and lifestyle stressors can shape physiological regulation, recovery capacity, and stress vulnerability. Accordingly, coping skills represent one component within a broader stress-management framework rather than being synonymous with it (Stanisławski, 2019; Ghasemi et al., 2024).

The most effective way to manage stress is to make it a daily training routine rather than a reactive response. Integrating stress-regulation strategies, including cognitive-behavioral and mindfulness-based approaches, into regular training has been demonstrated to mediate the association between life stress and athlete burnout, lower the occurrence of illness and injury, and facilitate more consistent performance throughout the season (Wilczyńska et al., 2022). These regular interventions, along with continuous observation of recovery using measures like heart-rate variability and sleep, can be used to identify dysregulation early and permit personalized manipulation of training load, recovery protocols, and psychological support before stress degenerates into burnout, illness, or injury (Jain et al., 2023; Hannon et al., 2025). This preventative strategy is further operationalised through wearable-based heart-rate variability and sleep monitoring systems, which offer real-world and continuous data, which can be used to manage stress in a timely and adaptive manner and ensure sustainable development of athletes.

### 3.3 One size doesn't fit all

Sport stress is complex and largely personalized and depends on the interplay of psychological, biological, social, and cultural factors; therefore, it does not appear in a similar manner in all athletes or in all sporting situations. There is always empirical evidence that shows universal stress-management programmes are restricted or even counterproductive because their effectiveness is modulated by the sport-specific load, age, competition level, gender, stage of development, and specific components of stress (Rumbold et al., 2012). Individualized models, such as psycho-neuro-endocrine-immunological ones, focus on flexible assessment, intervention, and monitoring, which are responsive to the sport, role, stage of the career, and the changing environment of the athlete (Tossici et al., 2024). Different sports and roles require different activation and recovery profiles; coping resources change across the athletic lifespan; gender-specific differences impact stress exposure and mental-health risk; and cultural contexts influence stress appraisal and recovery patterns (Nuetzel, 2025). In this regard, context-relevant and individualized stress-regulation programs are not only more effective but also morally aligned with athlete-centered care, which encourages engagement and minimizes the risk of unnecessary and maladaptive physical and psychological loads.

## 4 The IPP paradigm and its implementation

This paper presents the Integrative Preventive Personalized (IPP) paradigm, an evidence-based, conceptual, and practice-based framework proposed by the authors. The IPP has not been described here as a strictly validated intervention model; rather, it is a compilation of existing empirical evidence from sport psychology, psychophysiology, lifestyle medicine, and performance science, combined to address stress as a multisystem regulatory process. Although interventions such as heart rate variability–guided training, mindfulness-based stress reduction, yoga, lifestyle monitoring, and implicit coping development have each shown positive effects on stress regulation and wellbeing, the combined IPP framework has not yet been evaluated as a unified model. The empirical research conducted in the future should directly assess IPP as an integrated model. The IPP approach turns stress into a liability into a quantifiable, manageable, and highly operationalized construct, which facilitates high performance and wellness of athletes in the long run.

Structural change in sporting organizations is the key to the successful implementation of an IPP approach. Specifically, effective cross-functional teamwork and the strategic use of technology are major to its effectiveness. The support groups should be made to be more of an integrated unit where the psychologist, coach, medical staff, and trainers would share information both physiologically and psychologically in real time so that coordinated support guidelines can be developed. In addition, virtual-reality simulation and sophisticated biofeedback are integrated into training to ensure that safe environments are provided where implicit resilience is acquired with time. In this way, stress regulation becomes a coordinated, evidence-informed component of daily athletic preparation.

## 5 Conclusion

Stress is still being perceived by many as a mental, conscious and reactive phenomenon and this is a misconception that has always affected the wellbeing and performance of athletes. This shortsighted approach is still reflected in the management of stress in sports psychology. Currently, our system often treats stress as something to fight against only once it becomes problematic, which creates significant gaps and contributes to ongoing burnout. Rather than responding to stress, proactive approaches are to be taken. This new perspective views athletes as whole persons and puts an emphasis on integrative, preventive, and personalized approaches. Sports psychology can evolve into more sustainable athlete development by reframing stress as a multidimensional regulation process and adopting integrative, preventive and personalized strategies. This view acknowledges the interrelationships of mental, physiological, social, and cultural aspects of stress management. The strategies in stress management should also be person-centered and acknowledge the individuality of an athlete. Stress management can become a source of development, self-confidence, and success when implemented at an early stage and regularly.
